# 2-Fucosyllactose Metabolism by Bifidobacteria Promotes Lactobacilli Growth in Co-Culture

**DOI:** 10.3390/microorganisms11112659

**Published:** 2023-10-29

**Authors:** Alicja M. Nogacka, Isabel Cuesta, Miguel Gueimonde, Clara G. de los Reyes-Gavilán

**Affiliations:** 1 Department of Microbiology and Biochemistry of Dairy Products, Instituto de Productos Lácteos de Asturias (IPLA-CSIC), 33300 Villaviciosa, Asturias, Spain; icuesta@ipla.csic.es (I.C.); mgueimonde@ipla.csic.es (M.G.); greyes_gavilan@ipla.csic.es (C.G.d.l.R.-G.); 2 Institute of Health Research of the Principality of Asturias (ISPA), 33011 Oviedo, Asturias, Spain

**Keywords:** 2′FL, bifidobacteria, lactobacilli, degradation status, cross-feeding

## Abstract

Breastfeeding is recognized as the gold standard in infant nutrition, not only because of breastmilk’s intrinsic nutritional benefits but also due to the high content of different bioactive components such as 2-fucosyllactose (2′FL) in the mother’s milk. It promotes the growth of its two major consumers, *Bifidobacterium longum* ssp. *infantis* and *Bifidobacterium bifidum*, but the effect on other intestinal microorganisms of infant microbiota remains incompletely understood. pH-uncontrolled fecal cultures from infants donors identified as “fast 2′FL -degrader” microbiota phenotype were used for the isolation of 2′FL-associated microorganisms. The use of specific selective agents allowed the successful isolation of *B. bifidum* IPLA20048 and of *Lactobacillus gasseri* IPLA20136. The characterization of 2′FL consumption and its moieties has revealed more pronounced growth, pH drop, and lactic acid production after 2′FL consumption when both microorganisms were grown together. The results point to an association between *B. bifidum* IPLA20048 and *L. gasseri* IPLA20136 in which *L. gasseri* is able to use the galactose from the lactose moiety after the hydrolysis of 2′FL by *B. bifidum*. The additional screening of two groups of bifidobacteria (n = 38), fast and slow degraders of 2′FL, in co-culture with lactobacilli confirmed a potential cross-feeding mechanism based on degradation products released from bifidobacterial 2′FL break-down. Our work suggests that this phenomenon may be widespread among lactobacilli and bifidobacteria in the infant gut. More investigation is needed to decipher how the ability to degrade 2′FL and other human milk oligosaccharides could influence the microbiota establishment in neonates and the evolution of the microbiota in adult life.

## 1. Introduction

Breastfeeding is recognized as the gold standard in infant nutrition. Its exclusivity is the recommended feed habit for the infant during the first six months of life, with the continuation of breastfeeding up to 2 years of age [1]. This is not only because of breastmilk’s intrinsic nutritional benefits but is also due to the high content of different bioactive components in the mother’s milk. Some of them are the human milk oligosaccharides (HMOs) which represent the third-largest solid component of milk, after lactose and lipids, with a concentration ranging from 9 to 24 g/L. This group of carbohydrates is composed of about 200 different structures formed by fucosylated, sialylated, and neutral oligosaccharides with a lactose core at the reducing end, which vary in composition and concentration during lactation [2]. Additional variation in HMO structure can be largely linked to the maternal blood group, by the expression of Secretor and Lewis genes, encoding α1–2 fucosyltransferase (FUT2) and α1–4 fucosyltransferase (FUT3) enzymes, respectively [3]. The functional allele of FUT2 is used to classify mothers as “secretors”, and they produce 2-fucosyllactose (2′FL) as the most abundant HMO in milk. In contrast, “non-secretor” mothers produce mainly oligosaccharides with α-1,4- and α-1,3-fucosyl bonds [3]. Given that most women (about 75%) present a “secretor” phenotype [4], 2′FL may be regarded as the most abundant HMO. In addition, 2′FL is also commercially available, which attracts attention to the study of its metabolism by intestinal microbes.

HMOs may benefit the infant’s health in different ways, acting as antiadhesive antimicrobials in the gut, and as intestinal and immune cell modulators, favoring mineral absorption and influencing brain development [5]. HMOs are also well recognized by their prebiotic and “bifidogenic” effect: they are resistant to the upper gastrointestinal environment reaching the gut intact where they promote the selective growth of potentially beneficial bacteria, especially *Bifidobacterium* [6,7,8]. Serving as a selective substrate, HMOs modulate the gut microbiota composition by promoting beneficial bacteria and decreasing potential pathogens not able to consume it, such as members from *Clostridium*, *Enterococcus*, *Staphylococcus*, *Enterobacter*, *Cronobacter*, *Escherichia*, or *Salmonella* genera [9,10].

In particular, 2′FL has been reported to influence the infant microbiota composition by promoting the growth of its two major consumers, *Bifidobacterium longum* ssp. *infantis* and *Bifidobacterium bifidum* [11]. Other strains from *Bifidobacterium longum* subsp. *longum, Bifidobacterium dentium, Bifidobacterium kashiwanohense*, and *Bifidobacterium breve* species are also able to use 2′FL although to a minor extent [9,10,11,12,13]. Two different strategies for 2′FL degradation have been reported. *B. longum* subsp. *infantis* and *B. longum* subsp. *longum* tend to digest the sugar structure internally into the cell by its transportation through ATP-binding cassette (ABC) transporters. In contrast, *B. bifidum* breaks down 2′FL outside the cell by the action of extracellular 1,2-α-L-fucosidase enzymes and the subsequent release of fucose and lactose in the surrounding environment, which can either be left for the growth of other bacteria or transported internally to be metabolized inside the cell [6]. However, other intestinal microorganisms, which lack the exclusive HMO assimilation systems of bifidobacteria, have been reported to break down this HMO in pure culture, i.e., *Bacteroides fragilis*, *Bacteroides vulgatus*, and *Bacteroides thetaiotaomicron* or *Akkermansia muciniphila* [9,10,14]. However, the degradation of 2′FL by these microorganisms is less efficient and they are frequently outcompeted by bifidobacteria [6].

Intestinal microorganisms not able to utilize 2′FL can metabolize the building blocks of its structure, released by the primary degraders of this molecule together with some fermentative end-products, contributing to microbial cross-feeding interactions in the infant gut [6]. Most of these cross-feedings have been studied within the intestinal bifidobacterial community. A release of undigested 2′FL constituents by *B. bifidum* strains has been reported in mono-culture [15], HMO-supplemented fecal cultures [16], and *in vivo* from infant gut microbiome studies [17]. In this way, *B. bifidum* stimulated the growth of other *Bifidobacterium* species by releasing products of the partial degradation of HMOs unconsumed, and thereby increasing the prevalence of bifidobacteria in fecal communities. Similar findings were reported when four infant-derived bifidobacteria (*B. bifidum* R0071, *B. breve* M-16V, *B. infantis* R0033, and *B. infantis* M-63) were grown *in vitro* in the presence of 2′FL [18].

Besides the cross-feeding between bifidobacteria, synergistic interactions between 2′FL-consuming bifidobacteria and other gut microorganisms such as *Faecalibacterium prausnitzii* [19] or *Anaerobutyricum hallii* [20,21] have also been reported. In spite of this, the knowledge on synergistic mechanisms among bifidobacteria degrading 2′FL and other important commensal members of the intestinal microbiota of infants is still scarce. In a recent work, we found that in fecal cultures of formula-fed babies at two months of age in the presence of 2′FL, lactobacilli evolved to became dominant over a subdominant population of bifidobacteria [22]. In this context, we hypothesize that lactobacilli, which are not first degraders of 2′FL, could grow well on metabolites resulting from the degradation of 2′FL, favoring a possible interaction between lactobacilli and bifidobacteria to consume 2′FL. Taking this into account, the aim of the present work was to explore the interaction mechanisms of *Bifidobacterium* and *Lactobacillus* for the use of 2′FL.

## 2. Materials and Methods

### 2.1. Culture Media and Conditions

Carbohydrate-free semi-defined MRS [23] culture medium was used for the cultivation and initial isolation of bifidobacteria and lactobacilli strains from infant fecal cultures. The medium composition was as follows (gL^−1^): bacteriological peptone (Sigma, Madrid, Spain) 10.0, yeast extract (ThermoFisher Scientific, Bleiswijk, Netherlands) 5.0, sodium acetate (Sigma, Madrid, Spain) 5.0, ammonium citrate (VWR, Leuven, Belgium) 2.0, potassium phosphate (Merck, Darmstadt, Germany) 2.0, magnesium sulphate heptahydrate (VWR, Darmstadt, Germany) 0.366, manganese sulphate (Panreac, Barcelona, Spain) 0.05, and cysteine HCl (Acros Organics, Delphi, India) 2.5. The medium also included Tween 80 (Sigma, Madrid, Spain) 1 mL per liter. The medium was supplemented with different carbohydrate sources as indicated below. The pH was adjusted between 6.2 and 6.5 and the medium was autoclaved at 121 °C for 15 min. Selective and differential TOS-propionate (Merck, Darmstadt, Germany) and MRS-LP (MRS supplemented with lithium chloride 0.2% *w*/*v* and sodium propionate 0.3% *w*/*v*) agar culture plates were used for the isolation of single colonies of bifidobacteria, whereas MRS supplemented with bile at 0.3% *w*/*v* (MRS-bile) was used for lactobacilli selection [24]. All incubations were carried out at 37 °C in an anaerobic chamber (MG500, Don Whitley Scientific, West York-Shire, UK, with an 80% *v*/*v* N_2_, 10% *v*/*v* CO_2_, and 10% *v*/*v* H_2_ atmosphere), unless otherwise specified.

### 2.2. Isolation of Potential 2′FL-Degrader Microorganisms

pH-uncontrolled fecal cultures from three different infant donors (Table 1) identified as “fast 2′FL -degrader” microbiota phenotype [22], were performed in a basal culture medium designed specifically for the cultivation of infant feces (BMIF) [25]. BMIF was supplemented with 2′FL (Aequival^®^, Friesland Campina Ingredients, Paramus, NJ, USA) at 1% (*v*/*v*) as the unique carbon source with the objective of isolating 2′FL-degrading microorganisms. The fecal sample homogenization and fecal culture preparation were performed as described previously [22]. After 24 h of incubation, samples were plated on MRS-agar supplemented with 2′FL at 1% (*v*/*v*) and incubated for 24–72 h. Single colonies were picked, reisolated again in MRS-agar, and grown in MRS broth supplemented with 2′FL at 1% (*v*/*v*) in an attempt to obtain pure single-strain cultures. Frozen stocks were prepared and the strains were identified by partial amplification and sequencing of the 16S rRNA gene using primers plb16 and mlb16 as described elsewhere [26,27]. The breakdown of 2′FL by the isolated strains was confirmed by a second-round incubation in MRS broth with 2′FL (1%, *v*/*v*), with further determination of 2′FL consumption by HPLC, as described below. The end-point growth parameters of isolated strains in liquid medium were taken after 24 h of incubation in both rounds of incubation: pH (pHmeter SensION + PH3; HACH; Barcelona, Spain) and optical density (OD_600 nm_, Ultrospect 10 Cell Density Meter; Amersham Biosciences). Stocks of isolated colonies from the second-round incubation were submitted to a selective process of isolation of the constituent microorganisms in TOS-agar and MRS-LP for bifidobacteria and in MRS-bile in aerobic conditions for lactobacilli.

### 2.3. Co-Culture of B. bifidum IPLA20048 and L. gasseri IPLA20136 in the Presence of 2′FL and Its Moieties

Mono-cultures and co-cultures of *B. bifidum* IPLA20048 and *L. gasseri* IPLA20136, two strains isolated from infant fecal cultures in the present work, were performed in the previously described semi-defined liquid MRS supplemented at 0.3% (*v*/*v*) with 2′FL and with each of the constituents of 2′FL, namely glucose (Sigma, Madrid, Spain), galactose (Sigma, Madrid, Spain), fucose (Sigma, Madrid, Spain), and lactose (VWR, Leuven, Belgium). Experiments were performed in duplicate over two independent days, using negative controls without either carbon source or bacteria inoculum. Carbohydrates were freshly prepared the day of the experiment by diluting in MilliQ water at 10% (*w*/*v*) and sterilizing by filtration through a pore size of 0.45 µm. During the course of the microbial cultivation, samples were taken at the beginning (0 h), exponential (5 h), and stationary (24 h) phases of growth for the quantification of microorganisms by qPCR and determination of metabolites and sugars by HPLC. At the same sampling times, pH and OD_600 nm_ were also determined.

### 2.4. Quantification of B. bifidum and L. gasseri Species by qPCR

DNA was extracted from the harvested pellets of mono-cultures and co-cultures of *B. bifidum* and *L. gasseri* by using the Bacterial & Yeast Genomic kit (Eurx, Gdańsk, Poland), following the manufacturer’s instructions, and the isolated DNA was stored at −20 °C until use for qPCR analyses. Levels of *B. bifidum* and *L. gasseri* were determined at 0, 5, and 24 h of incubation in samples from the mono-cultures and co-cultures added with the different carbon sources by qPCR, using previously described primers and conditions [28,29]. Variations in the Log CFU/mL levels of the evaluated species were normalized by subtracting the basal growth in the negative control (MRS without carbon source) with the purpose of representing, as much as possible, the differences in growth in the carbohydrates tested and not due to other components of the culture medium.

### 2.5. Quantification of Carbohydrates and Metabolites by HPLC

The variations in the levels of 2′FL, lactose, and the monosaccharides glucose, galactose, and fucose, as well as organic acids formed during fermentation (lactic, acetic, and formic) were determined by HPLC. Cell-free supernatants collected from mono- and co-cultures were centrifuged at maximum speed at 4 °C and filtered (0.20 µm), and injected using an Alliance 2795 separation module by ion-exclusion chromatography through a column ICSep ICE-ION (Teknokroma Analitica, Barcelona, Spain). A PDA 2966 photodiode array detector was used for the determination and quantification of organic acids, a 2414 differential refractometer detector for determination and quantification of carbohydrates, and the Empower software (Walters, Milford, MA, USA) for the identification and quantification of peak areas. Chromatographic and analysis conditions were used as described elsewhere [22]. The results were expressed in mg/100 mL.

### 2.6. Screening of Co-Cultures from Different Strains of Bifidobacteria and Lactobacilli in the Presence of 2′FL 

Co-cultures combining different bifidobacteria and lactobacilli strains (final inoculum of each bacteria 0.1% *v*/*v*) from the microbial collection at IPLA-CSIC were carried out in semi-defined liquid MRS supplemented with 2′FL at a final concentration of 0.3% (*v*/*v*). Control reference cultures include mono-cultures of each microorganism in 2′FL, and in the absence of any carbon source added (negative control), and in the presence of glucose (0.3% *v*/*v*) (positive control). Single strains were previously reactivated in MRS-glucose plates, checked for uniform morphology, and propagated in MRS-glucose broth medium. On the day of the experiment, a washing step in pre-reduced PBS was followed by OD_600 nm_ adjustment to 1.0. Finally, bifidobacteria–lactobacilli mono-cultures and co-cultures were performed in 96-well plates in duplicate. The growth kinetics of each pair of the bifidobacteria–lactobacilli combination were obtained using a Microplate Reader Spectrophotometer (BioTek Instrument Inc., Winooski, VT, USA) by determining the OD_600 nm_ every 20 min. Additionally, initial pH and final pH, as well as end-point OD_600 nm_, were determined after a ½ dilution of cultures.

### 2.7. Statistical Analyses

The statistical analysis of results obtained was performed using the SPSS v.26 software (SPSS Inc., Chicago, IL, USA). All experiments were carried out in duplicate, including technical replicates in each experiment. The different comparisons were analyzed by univariate analysis of variance (ANOVA) and post hoc DMS test at each time of sampling to determine significant differences between the mono-cultures and co-cultures. Statistical significance was accepted as *p*-value < 0.05. Graphs were created using in PRISM v9.5.1 (GraphPad, La Jolla, CA, USA).

## 3. Results

### 3.1. Isolation and Identification of Microorganisms from Infant Gut Microbiota Associated with 2′FL Degradation

With the aim to unravel the potential role of intestinal lactobacilli in the metabolism of 2′FL, we isolated single colonies in MRS-2′FL agar from fecal cultures of babies previously identified with a “fast-2′FL -degrader” status, whose microbiotas were found enriched with the Lactobacillaceae family [22]. In a first attempt, 20 isolates were recovered from agar plates that have been identified as belonging to the following genera: *Enterococcus* (n = 3), *Staphylococcus* (n = 1), *Bifidobacterium* (n = 1), *Clostridium* (n = 1), plus 14 lactobacilli isolates from different species. The selection with 2′FL as the sole carbon source could be biased by the presence of yeast extract in the culture medium or by residues of other sugars present in 2′FL commercial preparations (87.1% purity). Therefore, a second round of incubation of the isolated and identified colonies was performed in MRS-2′FL broth, followed by the confirmation of 2′FL consumption by HPLC analyses. Of the initial 14 lactobacilli strains, only 8 reproduced the former observed growth (Table 1). From these eight isolates, a total 2′FL consumption was obtained for three of them; moreover, surprisingly, when these three isolates were submitted to a new round of identification, they rendered a different identification by the partial sequencing of the 16S rRNA compared to the first round, now being identified as *B. bifidum* (two strains) and *B. longum* (one strain). These results may suggest the existence of consortia between lactobacilli and bifidobacteria that can challenge their segregation in pure cultures.

**Figure 1:**
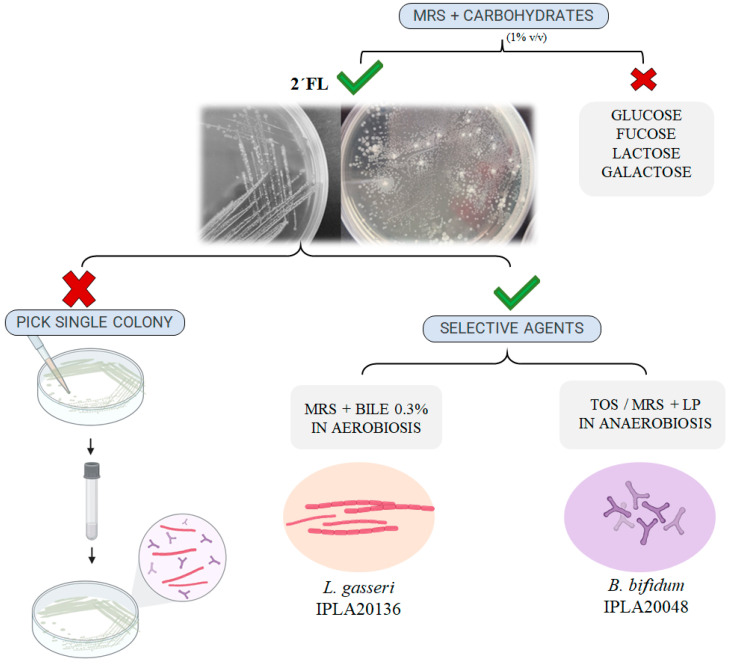
Schematic representation of strategies conducted to obtain isolates in pure cultures of *B. bifidum* IPLA20048 and *L. gasseri* IPLA20136 from a single colony obtained in MRS supplemented with 2′FL by spread plating the feces from infants with a 2′FL degrader phenotype. “LP” supplement containing lithium chloride 0.2% *w*/*v* and sodium propionate 0.3%, “TOS” Transgalactooligosaccharide-propionate agar.

In order to corroborate the hypothesis of an association between lactobacilli and bifidobacteria, to characterize the phenomenon, and to rule out possible contaminations during handling in the laboratory, we proceeded to the isolation of the lactobacilli and bifidobacteria potentially constituting the consortia suggested in Table 1. We first used the culture in MRS-2′FL which allowed us to distinguish different morphologies on agar plates (Figure 1). However, the attempt to pick single colonies with different morphologies and propagate the microorganisms in liquid and solid media was unsuccessful because both lactobacilli and bifidobacteria strains were always detected together when the presumptive isolated pure cultures were plated again in MRS-2′FL agar, and we checked for the presence of both microorganisms by PCR with specific primers or we observed them using an optical microscope. Then, we used different culture media with several selective agents. In this way, the use of MRS supplemented with LP and TOS allowed the successful isolation in pure culture from a single colony of a strain identified as *Bifidobacterium bifidum* (named strain IPLA20048), whereas the use of MRS supplemented with bile and incubated in aerobic conditions allowed the isolation in pure culture of *Lactobacillus gasseri* (named strain IPLA20136). New attempts of re-isolation from cultures of *B. bifidum* IPLA20048 and *L. gasseri* IPLA20136 and identification by partial 16S rRNA gene sequencing corroborated their single species identity.

### 3.2. Characterization of 2′FL -Consumption and Its Moieties in Co-Cultures of B. bifidum IPLA20048 and L. gasseri IPLA20136

We characterized co-cultures of the isolates *B. bifidum* IPLA20048 and *L. gasseri* IPLA20136 in liquid semi-defined MRS culture media supplemented either with 2′FL or each of its sugar constituents (lactose, fucose, galactose, and glucose), and determined the variations in the microbial levels and consumption of carbohydrates.

In the presence of 2′FL, after 24 h of incubation, co-cultures presented a significant increase in OD_600 nm_ and a more pronounced decrease in pH than mono-cultures of *L. gasseri* (*p*-value < 0.05, Table 2). In the remaining carbon sources tested, the co-culture condition resembled the microbial growth and the pH drops obtained in the mono-culture of *L. gasseri* (Table 2). These data suggest a potential interaction between *B. bifidum* IPLA20048 and *L. gasseri* IPLA20136 when 2′FL is present.

To obtain a deeper insight into the dynamics of this association, we quantified, by qPCR, the levels of bacteria in co-cultures by using specific primers for each species. Significantly higher increases in *L. gasseri* counts were found in co-cultures with respect to mono-cultures in all carbon sources tested (Table 2). In contrast, *B. bifidum* seems to maintain or slightly increase the levels achieved in mono-cultures only when co-cultures were incubated with 2′FL and lactose, whereas lower levels were reached when they were incubated with galactose or glucose.

**Figure 2:**
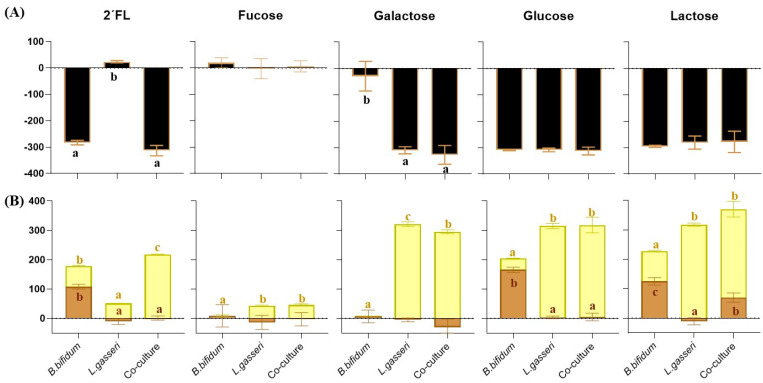
(**A**) Consumption of 2′FL and its constituents (Δmg/100 mL) and (**B**) variation in acetic (brown bars) and lactic acid (yellow bars) levels (Δmg/100 mL) after 24 h of incubation of *B. bifidum* IPLA20048 and *L. gasseri* IPLA20136 in mono-culture and co-culture. Significant differences between mono-cultures and co-cultures are indicated with different letters (*p*-value < 0.05).

Sugar and organic acid levels were quantified in supernatants collected from mono-cultures and co-cultures along incubation with the aim to study the metabolism of 2′FL and its constituent sugars with the two strains under study. The analysis of carbohydrate consumption at the end of incubation showed that *B. bifidum* was able to use 2′FL, lactose, and glucose but not galactose or fucose as a carbon source, whereas *L. gasseri* consumed all tested carbon sources except 2′FL and fucose (Figure 2A).

After 24 h of incubation, we obtained increases in acetic and lactic acids in *B. bifidum* IPLA 20048 mono-cultures and increases in lactic acid concentration in mono-cultures of *L. gasseri* IPLA 20136 (Figure 2B; Supplementary Table S1). Analyzing in detail the co-cultures as compared to mono-cultures, *L. gasseri* displayed a faster fermentation ability than *B. bifidum* (Supplementary Table S1), especially in glucose, and the metabolism of this microorganism is dominant in galactose, where *B. bifidum* was not able to grow, producing high levels of lactic acid. Interesting results were obtained in cultures with 2′FL after fermentation. Although *L. gasseri* grown in mono-culture produced low amounts of lactic acid, an increase in the production of this compound occurred in co-culture, pointing to an enhancement in the metabolism of *L. gasseri* in co-culture with respect to mono-culture (*p*-value < 0.05). Remarkably, in co-cultures of *B. bifidum* and *L. gasseri* with 2′FL and lactose, despite the growth of *B. bifidum* not being affected by the presence of *L. gasseri*, significantly lower levels of acetic acid were detected as compared with the mono-culture of *B. bifidum* (Figure 2B).

Our results indicate that *L. gasseri* benefits from the association, whereas *B. bifidum* maintains its levels by comparing the condition of mono-culture with respect to co-cultures. This is in contrast to that occurring with the other carbon sources tested where a decrease in the bifidobacteria was found when co-cultured with the lactobacilli. In short, the presence of *B. bifidum* IPLA 20048 promotes the proliferation of *L. gasseri* IPLA20136 when 2′FL is available, allowing *L. gasseri* to grow in a substrate that otherwise this microorganism would not be able to use.

### 3.3. Screening and Clustering of Bifidobacteria Strains by Their Ability to Use 2′FL and Behavior in Co-Cultures with Lactobacilli

With the aim of exploring whether similar associations as that of *B. bifidum* IPLA 20048 and *L. gasseri* IPLA 20136 could exist among other bifidobacteria and lactobacilli, we carried out mono-cultures and co-cultures combining 38 bifidobacteria strains (including commercial strains and isolates from babies, adults, and elderlies; Supplementary Table S2) and three lactobacilli, unable to use 2′FL, isolated from infant feces (*L. gasseri* IPLA 20136, *L. gasseri* IPLA20216, and *Lacticaseibacillus paracasei* IPLA20124) in MRS semi-defined broth supplemented with 2′FL and compared to positive (glucose as the sole carbon source) and negative controls (no carbon source added), by monitoring growth (OD_600 nm_) over 24 h of incubation.

An initial grouping of bifidobacteria strains according to their capacity to use 2′FL was carried out based on their growth kinetics and acidification in comparison with the positive control (Supplementary Table S2). Strains were considered as “2′FL -degrader” when OD_600 nm_ and the decrease in pH were similar or higher than those obtained in glucose. In this way, the 2′FL degradation phenotype was detected only in strains belonging to *B. bifidum* and *B. longum* species, but not in those strains belonging to *Bifidobacterium adolescentis*, *B. breve, Bifidobacterium animalis*, and *Bifidobacterium catenulatum/pseudocatenulatum* species (Table 3).

**Figure 3:**
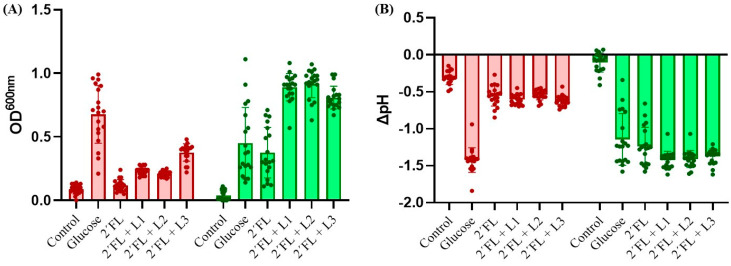
Growth of 2′FL-non-degrader bifidobacteria (red bars, n = 19), and 2′FL-degrader bifidobacteria (green bars, n = 19) in mono-cultures and co-cultures with three selected lactobacilli strains in the presence of 2′FL. (**A**) Optical density (OD_600 nm_) and (**B**) pH decrease after 24 h of incubation. “L1” *L. gasseri* IPLA20136; “L2” *L. gasseri* IPLA20126; ”L3” *L. paracasei* IPLA20124. “Control”: mono-culture of bifidobacteria without added carbon source.

The vast majority of 2′FL-degrader bifidobacteria were *B. bifidum* strains (13 out of 19 strains) and all of them presented faster growth in 2′FL than in glucose (Supplementary Figure S1, Supplementary Table S2). An age-specific association in the 2′FL-degrader phenotype was not found, as 2′FL-degrader *B. bifidum* strains were also isolated from the feces of adults and elderlies and not just from infant fecal samples. Clear enhanced growth (OD_600 nm_) and higher acidification (decrease of pH) were obtained after 24 h of incubation when 2′FL-degrader *Bifidobacterium* strains were co-cultured with the three lactobacilli strains in the presence of 2′F (*p*-value < 0.05) (Figure 3). This enhancement in growth was not obtained when bifidobacteria were not 2′FL degraders.

Our results point to a cross-feeding mechanism between *B. bifidum* IPLA20048 *and L. gasseri* IPLA20136 in which *L. gasseri* IPLA20136 could be able to use the galactose released from the lactose moiety after the hydrolysis of 2′FL by *B. bifidum* IPLA20048. Furthermore, the results of co-culturing 38 strains of bifidobacteria with three lactobacilli strains suggest that this association based on the breakdown of 2′FL could be widespread among fast-degrader bifidobacteria and intestinal lactobacilli.

## 4. Discussion

In a previous work addressing the influence of 2′FL on the infant’s gut microbiota, we identified specific microbial profiles depending on the intrinsic ability of the microbiota to degrade 2′FL and the mode of feeding (breastfed or formula-fed) [22]. We found that the consumption of 2′FL was associated with a significant increase in the Lactobacillaceae family, especially in formula-fed infants in fecal cultures. Similar lactobacilli increases were observed by other authors in infant fecal cultures with 2′FL [30]. Also, the infant’s ability to metabolize different HMOs has been associated with a higher relative abundance of lactobacilli in feces [31]. Nevertheless, the association of intestinal lactobacilli with HMOs is not well understood, especially because unlike bifidobacteria, the capacity of lactobacilli to use HMOs and to hydrolyze terminal sugars is rather limited [32,33].

**Figure 4:**
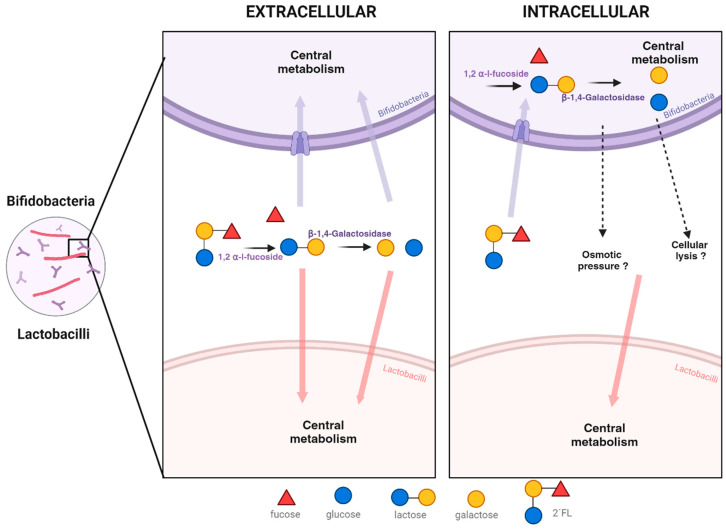
Intracellular and extracellular 2′FL degradation by bifidobacteria and its potential impact on lactobacilli growth in co-culture.

The mechanisms by which 2′FL undergo bifidobacterial degradation can be carried out intracellularly by *B. longum* (transport-dependent) and extracellularly by *B. bifidum* (glycosidase-dependent) [11,13,34,35,36,37] (Figure 4). In the case of an extracellular mechanism, *B. bifidum* glycosidase enzymes can release galactose and lactose to the milieu, making them readily available for lactobacilli growth. This tendency to accumulate degradation products such as galactose, fucose, or lactose was described in several *B. bifidum* strains after incubation in the presence of HMOs [15,16,38]. The extracellular activity of *B. bifidum* on structurally similar oligosaccharides found in mucin was already described accompanied by a release of galactose, and subsequent uptake by co-cultured microorganisms such as *A. hallii* or *B. breve* [39,40]. In the case of the bifidobacterial intracellular mechanism of 2′FL degradation, there is not a clear explanation on how the 2′FL moieties become available for lactobacilli growth promotion. Still, other authors detected a transient increase in the levels of monosaccharides and lactose in supernatants from the fermentation of HMOs by *B. longum* or *B. infantis*, in spite of the intracellular location of the glycosidase activity [8,15,41]. A possible explanation for this phenomenon could be the transport outside the cell of saccharides generated internally from the hydrolysis of HMOs to counteract changes in the osmotic pressure caused by the rapid intracellular incorporation of HMOs, or by the cellular lysis of bifidobacteria (Figure 4) [8,15,41], although the hypothetic cell transport mechanisms remain unknown.

While lactobacilli benefit from the association with bifidobacteria by obtaining a carbon and energy source, the benefit of bifidobacteria in this association is unclear. Centanni and co-workers co-cultivated a *B. bifidum* 2′FL-degrader with a 2′FL-non-degrader *B. breve* strain which resulted in an enhancement in the growth of *B. breve*, remaining the abundance of the *B. bifidum* degrading strain at the same levels in mono-culture and co-culture [42]. The transcriptomic analysis of co-cultures revealed upregulated genes encoding α-fucosidase, resulting in more lactose becoming available. It seems that this adjustment could allow *B. bifidum* to maintain growth and be more active metabolically by increasing gene transcription involved in carbohydrate transport, and energy production and conversion. This discovered association in the context of 2′FL impact on infant microbiota can be explained by the “Black Queen hypothesis”. This theory hypothesizes that the donor/2′FL-degrader microorganism and the subsequent beneficiary species compete for released substances, with the critical feature of this association being the donor´s function in favor of “public good” by producing substances for use by others, allowing them to continue growing together in the community [42]. In another study, a similar phenomenon was observed when four infant-derived bifidobacterial strains were co-cultured in the presence of 2′FL (*B. bifidum* R0071, *B. breve* M-16V, *B. infantis* R0033, and *B. infantis* M-63) [18], in which no increases in bacterial cell numbers were found for the 2′FL-degrading bifidobacteria strains. These results seem to provide a metabolic explanation for the distribution of bifidobacterial species in the gut of breast-fed infants. Classical HMO consumers *B. bifidum* and *B. infantis* are often detected in low numbers in the feces of breast-fed infants, while *B. longum* and *B. breve* are regularly found as the dominant species in infant stools, even though they demonstrate minimal growth in HMO *in vitro* [8,13,18,43]. In spite of that, these types of associations may bring some benefit for 2′FL-degrading bifidobacteria, such as the above-mentioned upregulation of bifidobacterial carbohydrate transporters [42,44], or syntrophic interactions based on the end-products of HMO metabolism such as 1,2-propanediol, and acetic and lactic acid metabolized to propionate or butyrate by other gut members [20]. More investigation is needed about the interaction between 2′FL-degrading bifidobacteria and other members of the gut microbiota, and how the ability to degrade this and other HMO could influence the microbiota establishment in neonates and the evolution of the microbiota in adult life.

## 5. Conclusions

A consortium between bifidobacteria and lactobacilli was identified in fecal cultures of infants with a “fast-2’FL-degrader” status of their gut microbiota. The use of specific selective agents allowed the successful isolation, in pure culture, of *B. bifidum* IPLA20048 and of *L. gasseri* IPLA20136. Based on the characterization of 2’FL consumption and its components, it was evidenced that the presence of both bifidobacteria and lactobacilli in co-culture contributed to enhance microbial growth, pH decrease, and lactic acid production concomitantly with 2’FL consumption. This finding contrasts other tested carbohydrates where the co-culture of bifidobacteria and lactobacilli resembled lactobacilli growth in monoculture. Our results suggest a cross-feeding between *B. bifidum* IPLA20048 and *L. gasseri* IPLA20136, in which *L. gasseri* utilizes galactose from a lactose moiety after 2′FL hydrolysis by *B. bifidum*. The additional screening of co-cultures with other bifidobacteria and lactobacilli strains from different origins suggests that the phenomenon of cross-feeding based on products released from bifidobacterial 2′FL degradation may be widespread in the infant gut.

## Figures and Tables

**Table 1:** Identification by amplification and partial sequencing of the 16S rRNA gene of lactobacilli isolated colonies obtained from fecal cultures after 24 h of incubation in MRS supplemented with 2′FL (1%, *v*/*v*) (First identification) and after a second round of incubation of the same purified colonies (Second Identification). 2′FL consumption (%), optical density (OD_600 nm_), and pH were determined at the end of incubation (Second Identification). The shaded event was submitted to the isolation of the microorganisms in selective culture media, followed by the characterization of the interaction among the identified strains, named as *B. bifidum* IPLA20048 and *L. gasseri* IPLA20136.

Infant	Feeding	Gender	Birth	Age (Months)	First Identification	Second Identification	2′FL Consumption	OD_600 nm_	pH
1	Exclusive Formula	Male	Vaginal	2	*Lactobacillus para/gasseri*	*Lactobacillus para/gasseri*	0.31	0.53	5.89
					*Lactobacillus para/gasseri*	***Bifidobacterium bifidum***	**−100.00**	1.70	4.65
2	Exclusive Formula	Male	Vaginal	2	*Lactobacillus para/gasseri*	*Lactobacillus para/gasseri*	2.88	0.60	5.80
					*Lactobacillus para/gasseri*	*Lactobacillus para/gasseri*	1.58	0.60	5.84
					*Lacticaseibacillus rhamnosus/casei/*	*Lacticaseibacillus rhamnosus*	9.78	0.98	6.00
					*Limosilactobacillus reuteri/balticus/agrestis*	*Limosilactobacillus balticus/agrestis/reuteri*	−0.59	1.01	6.27
3	Formula Breast milk	Male	Vaginal	2	*Lactobacillus vaginalis*	***Bifidobacterium longum***	**−99.93**	3.94	4.16
					*Lactobacillus* spp.	***Bifidobacterium bifidum***	**−100.00**	2.68	4.54

**Table 2:** Microbial growth (OD_600 nm_ and pH decreases over time) and microbial levels (increases over time, ∆Log CFU/mL) of mono-cultures and co-cultures of *B. bifidum* IPLA20048 and *L. gasseri* IPLA20136 in MRS supplemented with carbohydrate constituent of 2′FL after 24 h of incubation. Significant differences between mono- and co-cultures are indicated with different letters (*p*-value < 0.05).

	Parameters	Carbon Source	*B. bifidum*	*L. gasseri*	Co-Culture	*p*-Value
Microbial growth	OD_600 nm_	2′FL	0.99 ± 0.32 ^ab^	0.57 ± 0.08 ^a^	1.68 ± 0.12 ^b^	**0.024**
		Fucose	0.13 ± 0.12 ^a^	0.42 ± 0.10 ^b^	0.51 ± 0.10 ^b^	**0.028**
		Galactose	0.37 ± 0.26 ^a^	1.91 ± 0.13 ^b^	1.92 ± 0.02 ^b^	**0.009**
		Glucose	1.01 ± 0.19 ^a^	1.61 ± 0.11 ^b^	1.68 ± 0.10 ^b^	**0.035**
		Lactose	1.15 ± 0.23 ^a^	1.89 ± 0.03 ^b^	1.91 ± 0.03 ^b^	**0.001**
		Control	0.23 ± 0.10 ^a^	0.73 ± 0.10 ^b^	0.79 ± 0.08 ^b^	**0.016**
	∆ pH	2′FL	−1.09 ± 0.11 ^a^	−0.23 ± 0.12 ^b^	−1.12 ± 0.15 ^a^	**0.016**
		Fucose	−0.02 ± 0.06	−0.16 ± 0.08	−0.26 ± 0.04	0.252
		Galactose	−0.21 ± 0.26 ^b^	−1.27 ± 0.06 ^b^	−1.26 ± 0.06 ^b^	**0.006**
		Glucose	−1.32 ± 0.08	−1.38 ± 0.04	−1.35 ± 0.02	0.704
		Lactose	−0.96 ± 0.53	−1.40 ± 0.11	−1.37 ± 0.08	0.896
		Control	0.11 ± 0.18	−0.32 ± 0.10	−0.30 ± 0.03	0.056
Microbial levels Log (UFC/mL)	∆ *B. bifidum*	2′FL	1.74 ± 0.16 ^b^	0.29 ± 0.41 ^a^	1.64 ± 0.08 ^b^	**0.019**
		Fucose	−0.34 ± 0.64	0.18 ± 0.25	0.36 ± 0.14	0.335
		Galactose	0.86 ± 0.50 ^b^	0.00 ± 0.00 ^a^	0.64 ± 0.10 ^b^	**0.001**
		Glucose	2.72 ± 0.86 ^b^	0.00 ± 0.00 ^a^	0.78 ± 0.56 ^a^	**0.041**
		Lactose	1.96 ± 0.49 ^b^	0.00 ± 0.00 ^a^	1.62 ± 0.30 ^b^	**0.019**
	∆ *L. gasseri*	2′FL	−0.05 ± 0.10 ^a^	−0.08 ± 0.21 ^a^	2.35 ± 0.03 ^b^	**0.001**
		Fucose	−0.14 ± 0.19 ^a^	−0.09 ± 0.21 ^a^	0.75 ± 0.25 ^b^	**0.044**
		Galactose	−0.14 ± 0.19 ^a^	1.17 ± 0.57 ^b^	2.33 ± 0.14 ^c^	**0.014**
		Glucose	−0.14 ± 0.19 ^a^	1.35 ± 0.14 ^b^	2.28 ± 0.36 ^c^	**0.005**
		Lactose	−0.14 ± 0.19 ^a^	1.43 ± 0.55 ^b^	2.20 ± 0.13 ^b^	**0.014**

**Table 3:** *Bifidobacterium* strains from different origins used in co-culture with lactobacilli, grouped by their ability to grow in 2′FL. “B.bif” *B.bifidum*; “B.long” *B.longum*; “B.adol” *B.adolescentis*; “B.brev” *B. breve*; “B.p/cate” *B.pseudocatenulatum* or *B.catenulatum*.

Phenotype	Origin	B.bif	B.long	B.adol	B.brev	B.anim	B.p/cate	Total
2′FL-degrader	Infants	5	4					19
	Adults	1						
	Obese	1						
	Elderly	4	2					
	Commercial	2						
Non-degrader	Infants	1	3		4	2	3	19
	Adults			1				
	Obese		1					
	Elderly		1				3	
	Commercial							

## Data Availability

Additional data presented in this study are available upon reasonable request from the corresponding author.

## Supplementary Materials

The following supporting information can be downloaded at https://www.mdpi.com/article/10.3390/microorganisms11112659/s1, Figure S1: Kinetics growth curves along 24 h of incubation of 38 tested *Bifidobacterium* strains with glucose (red lines), 2’FL (green lines), and negative control (grey lines) classified by capability of 2′FL degradation.; Table S1: Variation in time (after 5 and 24 h) of carbohydrate and organic acid levels (mg/100 mL) in mono-cultures and co-cultures of *B. bifidum* IPLA20048 and *L. gasseri* IPLA20136 in MRS supplemented with carbohydrate constituents of 2’FL.; Table S2: End-point growth parameters of OD_600 nm_ and pH after 24 h of incubation with 2’FL and respective negative (PBS) and positive controls (glucose) of 38 bifidobacteria strains classified by 2’FL status degradation.
