# Supplementary material for: 2-Fucosyllactose Metabolism by Bifidobacteria Promotes Lactobacilli Growth in Co-Culture
Source: Microorganisms. 2023 Oct 29;11(11):2659. doi: 10.3390/microorganisms11112659 (PMC10673426; doi:10.3390/microorganisms11112659)
Supplement: Supplementary file 1 [file microorganisms-11-02659-s001.zip › Supplementary Table S1.pdf]

Supplementary Table S1. Variation in time (after 5 and 24 hours) of carbohydrates and organic acids levels (mg/100mL) in mono-cultures and co-cultures of *B.bifidum* IPLA20048 and *L.gasseri* IPLA20136 in MRS supplemented with carbohydrates constituent of 2´FL.

		5				24			
		B.bifidum	*L.gasseri*	Co-culture	*p*-value	*B.bifidum*	*L.gasseri*	Co-culture	*p*-value
	**Sugar**								
2´FL	Δ 2FL	-43.13 ± 22.41	26.10 ± 18.05	-71.52 ± 56.46	0.152	**-283.04 ± 8.59^{a}**	**23.67 ± 4.38^{b}**	**-313.23 ± 19.77^{a}**	**0.000**
	Δ lactose	0.00 ± 0.00	0.00 ± 0.00	0.00 ± 0.00		0.00 ± 0.00	0.00 ± 0.00	0.00 ± 0.00	
	Δ glucose	**-0.43 ± 0.65^{b}**	**-2.69 ± 0.34^{a}**	**-3.49 ± 0.09^{a}**	**0.012**	**-1.41 ± 0.20^{c}**	**-2.69 ± 0.34^{b}**	**-3.49 ± 0.09^{a}**	**0.007**
	Δ galactose	**2.09 ± 0.22^{b}**	**-0.97 ± 0.20^{a}**	**-0.79 ± 0.52^{a}**	**0.005**	**8.25 ± 1.51^{b}**	**-3.12 ± 0.06^{a}**	**-3.61 ± 0.34^{a}**	**0.002**
	Δ fucose	23.20 ± 11.06	0.00 ± 0.00	25.08 ± 13.96	0.155	**95.97 ± 0.59^{b}**	**0.00 ± 0.00^{a}**	**93.89 ± 4.71^{b}**	**0.000**
	**Organic acids**								
	Δ lactic acid	**4.76 ± 2.88^{a}**	**48.26 ± 2.58^{b}**	**65.69 ± 9.83^{b}**	**0.005**	**70.41 ± 2.98^{b}**	**50.96 ± 0.79^{a}**	**216.30 ± 1.47^{c}**	**0.000**
	Δ formic acid	0.00 ± 0.00	0.00 ± 0.00	0.00 ± 0.00		**6.47 ± 0.24^{b}**	**0.00 ± 0.00^{a}**	**0.00 ± 0.00^{a}**	**0.000**
	Δ acetic acid	17.38 ± 11.59	6.14 ± 22.10	-3.29 ± 17.33	0.565	**107.95 ± 8.08^{b}**	**-10.54 ± 10.03^{a}**	**1.12 ± 7.05^{a}**	**0.001**
	**Sugar**								
Fucose	Δ 2FL	0.00 ± 0.00	0.00 ± 0.00	0.00 ± 0.00		0.00 ± 0.00	0.00 ± 0.00	0.00 ± 0.00	
	Δ lactose	0.00 ± 0.00	0.00 ± 0.00	0.00 ± 0.00		0.00 ± 0.00	0.00 ± 0.00	0.00 ± 0.00	
	Δ glucose	**-0.83 ± 0.49^{b}**	**-2.30 ± 0.11^{a}**	**-2.92 ± 0.09^{a}**	**0.012**	**-1.11 ± 0.45^{b}**	**-2.30 ± 0.11^{a}**	**-2.92 ± 0.09^{a}**	**0.015**
	Δ galactose	0.19 ± 0.56	-0.64 ± 0.35	-0.51 ± 0.26	0.240	**0.56 ± 0.34^{b}**	**-2.73 ± 0.14^{a}**	**-2.85 ± 0.51^{a}**	**0.004**
	Δ fucose	29.12 ± 9.05	5.12 ± 23.04	-10.01 ± 28.68	0.332	20.03 ± 18.32	-1.87 ± 38.10	6.47 ± 21.07	0.743
	**Organic acids**								
	Δ lactic acid	**1.23 ± 1.63^{a}**	**42.84 ± 1.92^{b}**	**40.16 ± 3.63^{b}**	**0.001**	**0.31 ± 2.63^{a}**	**42.40 ± 2.64^{b}**	**45.93 ± 3.78^{b}**	**0.001**
	Δ formic acid	0.00 ± 0.00	0.00 ± 0.00	0.00 ± 0.00		0.00 ± 0.00	0.00 ± 0.00	0.00 ± 0.00	
	Δ acetic acid	21.81 ± 25.37	2.25 ± 9.55	-12.55 ± 31.53	0.456	8.76 ± 38.03	-13.89 ± 24.06	-3.21 ± 22.40	0.758
	**Sugar**								
Galactose	Δ 2FL	0.00 ± 0.00	0.00 ± 0.00	0.00 ± 0.00		0.00 ± 0.00	0.00 ± 0.00	0.00 ± 0.00	
	Δ lactose	0.00 ± 0.00	0.00 ± 0.00	0.00 ± 0.00		0.00 ± 0.00	0.00 ± 0.00	0.00 ± 0.00	
	Δ glucose	**-0.98 ± 0.19^{b}**	**-2.01 ± 0.21^{a}**	**-2.52 ± 0.22^{a}**	**0.011**	**-1.03 ± 0.03^{b}**	**-2.01 ± 0.21^{a}**	**-2.52 ± 0.22^{a}**	**0.008**
	Δ galactose	3.15 ± 17.90	-26.72 ± 20.21	-79.06 ± 88.63	0.412	**-29.11 ± 56.27^{b}**	**-310.06 ± 13.59^{a}**	**-327.54 ± 35.67^{a}**	**0.008**
	Δ fucose	0.00 ± 0.00	0.00 ± 0.00	0.00 ± 0.00		0.00 ± 0.00	0.00 ± 0.00	0.00 ± 0.00	
	**Organic acids**								
	Δ lactic acid	-1.06 ± 0.29	101.59 ± 36.36	105.91 ± 38.56	0.064	**1.12 ± 0.18^{a}**	**321.38 ± 8.04^{c}**	**295.72 ± 6.77^{b}**	**0.000**
	Δ formic acid	0.00 ± 0.00	0.00 ± 0.00	0.00 ± 0.00		9.00 ± 6.37	0.00 ± 0.00	0.00 ± 0.00	0.143
	Δ acetic acid	-13.59 ± 9.08	19.56 ± 0.71	-9.41 ± 31.12	0.298	6.71 ± 21.52	-4.81 ± 6.27	-30.33 ± 28.12	0.326
	**Sugar**								
Glucose	Δ 2FL	0.00 ± 0.00	0.00 ± 0.00	0.00 ± 0.00		0.00 ± 0.00	0.00 ± 0.00	0.00 ± 0.00	
	Δ lactose	0.00 ± 0.00	0.00 ± 0.00	0.00 ± 0.00		0.00 ± 0.00	0.00 ± 0.00	0.00 ± 0.00	
	Δ glucose	**0.86 ± 5.15^{b}**	**-270.82 ± 45.99^{a}**	**-276.29 ± 65.80^{a}**	**0.015**	-308.72 ± 2.65	-308.21 ± 6.90	-313.03 ± 14.72	0.863
	Δ galactose	0.36 ± 0.75	2.59 ± 0.82	0.94 ± 3.14	0.553	1.55 ± 0.45	-1.08 ± 1.52	-2.35 ± 0.41	0.056
	Δ fucose	0.00 ± 0.00	0.00 ± 0.00	0.00 ± 0.00		0.00 ± 0.00	0.00 ± 0.00	0.00 ± 0.00	
	**Organic acids**								
	Δ lactic acid	1.44 ± 1.05	269.04 ± 47.69	259.14 ± 45.79	0.010	**38.70 ± 1.27^{a}**	**312.26 ± 8.46^{b}**	**313.22 ± 26.81^{b}**	**0.001**
	Δ formic acid	0.00 ± 0.00	0.00 ± 0.00	0.00 ± 0.00		**30.25 ± 1.33^{b}**	**0.00 ± 0.00^{a}**	**0.00 ± 0.00^{a}**	**0.000**
	Δ acetic acid	10.18 ± 5.30	6.34 ± 1.21	-7.72 ± 6.71	0.072	**165.67 ± 8.87^{b}**	**3.49 ± 4.41^{a}**	**4.97 ± 12.98^{a}**	**0.001**
	**Sugar**								
Lactose	Δ 2FL	0.00 ± 0.00	0.00 ± 0.00	0.00 ± 0.00		0.00 ± 0.00	0.00 ± 0.00	0.00 ± 0.00	
	Δ lactose	-33.22 ± 17.96	-18.63 ± 21.93	-60.57 ± 42.82	0.456	-295.15 ± 4.01	-280.98 ± 24.74	-278.15 ± 40.47	0.815
	Δ glucose	1.73 ± 2.53	-2.39 ± 0.28	0.28 ± 3.38	0.361	**-1.46 ± 0.01^{b}**	**-2.39 ± 0.28^{a}**	**-2.81 ± 0.17^{a}**	**0.013**
	Δ galactose	8.68 ± 3.43	-1.82 ± 0.29	2.30 ± 4.10	0.092	**25.32 ± 6.34^{b}**	**-2.90 ± 0.11^{a}**	**-3.28 ± 0.38^{a}**	**0.007**
	Δ fucose	0.00 ± 0.00	0.00 ± 0.00	0.00 ± 0.00		0.00 ± 0.00	0.00 ± 0.00	0.00 ± 0.00	
	**Organic acids**								
	Δ lactic acid	**8.13 ± 1.87^{a}**	**49.62 ± 4.28^{b}**	**72.71 ± 8.68^{c}**	**0.003**	**102.65 ± 2.74^{a}**	**318.25 ± 4.91^{b}**	**300.69 ± 26.84^{b}**	**0.001**
	Δ formic acid	0.00 ± 0.00	0.00 ± 0.00	0.00 ± 0.00		**9.34 ± 1.26^{b}**	**0.00 ± 0.00^{a}**	**0.00 ± 0.00^{a}**	**0.002**
	Δ acetic acid	8.76 ± 3.85	-6.40 ± 23.07	13.31 ± 20.64	0.580	**125.52 ± 12.55^{c}**	**-10.89 ± 12.11^{a}**	**70.02 ± 15.68^{b}**	**0.005**

	**Sugar**								
Control	Δ 2FL	0.00 ± 0.00	0.00 ± 0.00	0.00 ± 0.00		0.00 ± 0.00	0.00 ± 0.00	0.00 ± 0.00	
	Δ lactose	0.00 ± 0.00	0.00 ± 0.00	0.00 ± 0.00		0.00 ± 0.00	0.00 ± 0.00	0.00 ± 0.00	
	Δ glucose	**-0.60 ± 0.19[b]**	**-2.17 ± 0.00[a]**	**-2.51 ± 0.54[a]**	**0.020**	-0.86 ± 0.48	-2.17 ± 0.00	-2.51 ± 0.54	0.056
	Δ galactose	0.35 ± 0.17	-0.01 ± 1.47	-0.55 ± 0.62	0.664	0.32 ± 0.39	-1.97 ± 1.70	-2.65 ± 0.90	0.152
	Δ fucose	0.00 ± 0.00	0.00 ± 0.00	0.00 ± 0.00		0.00 ± 0.00	0.00 ± 0.00	0.00 ± 0.00	
	**Organic acids**								
	Δ lactic acid	**0.65 ± 0.95[a]**	**41.91 ± 3.28[b]**	**41.50 ± 2.28[b]**	**0.001**	**0.36 ± 0.12[a]**	**44.22 ± 1.83[b]**	**46.55 ± 2.92[b]**	**0.000**
	Δ formic acid	0.00 ± 0.00	0.00 ± 0.00	0.00 ± 0.00		0.00 ± 0.00	0.00 ± 0.00	0.00 ± 0.00	
	Δ acetic acid	7.63 ± 16.89	-3.83 ± 23.34	7.69 ± 23.90	0.837	6.48 ± 8.40	-14.93 ± 10.68	16.16 ± 25.88	0.310
