# Supplementary material for: 2-Fucosyllactose Metabolism by Bifidobacteria Promotes Lactobacilli Growth in Co-Culture
Source: Microorganisms. 2023 Oct 29;11(11):2659. doi: 10.3390/microorganisms11112659 (PMC10673426; doi:10.3390/microorganisms11112659)
Supplement: Supplementary file 1 [file microorganisms-11-02659-s001.zip › Supplementary Table S2.pdf]

Supplementary Table S2. End-point growth parameters of OD_{600nm} and pH after 24 hours of incubation with 2´FL and respective negative (PBS) and positive controls (glucose) of 38 bifidobacteria strains classified by 2´FL status degradation. "F" Female; "M" Male; "NA" no data

					OD_{600nm}			pH		
**Strain**	**Origin**	**Donor Gender**	**Identification**	**Main characteristics of growth**	**C-**	**2´FL**	**C+**	**C-**	**2´FL**	**C+**
**Non degraders**										
IPLA20044	Adults	F	*Bifidobacterium adolescentis*		0.07 ± 0.00	0.10 ± 0.00	0.58 ± 0.04	6.23 ± 0.02	5.90 ± 0.00	4.99 ± 0.01
IPLA20046	Adults	F	*Bifidobacterium longum*		0.03 ± 0.01	0.05 ± 0.01	0.37 ± 0.04	6.19 ± 0.01	5.62 ± 0.16	4.96 ± 0.00
IPLA20020	Babies	M	*Bifidobacterium animalis subsp. lactis*		0.06 ± 0.00	0.07 ± 0.01	0.92 ± 0.06	6.06 ± 0.06	5.75 ± 0.12	4.84 ± 0.10
IPLA20021	Babies	M	*Bifidobacterium animalis subsp. lactis*		0.10 ± 0.01	0.13 ± 0.01	0.95 ± 0.07	6.06 ± 0.05	5.82 ± 0.11	4.99 ± 0.01
IPLA20016	Babies	M	*Bifidobacterium bifidum*		0.00 ± 0.00	0.08 ± 0.08	0.21 ± 0.02	6.07 ± 0.02	6.11 ± 0.13	5.44 ± 0.02
IPLA20004	Babies	M	*Bifidobacterium breve*		0.10 ± 0.03	0.12 ± 0.02	0.87 ± 0.53	6.00 ± 0.13	5.71 ± 0.14	4.84 ± 0.11
IPLA20005	Babies	M	*Bifidobacterium breve*		0.14 ± 0.08	0.15 ± 0.05	0.71 ± 0.06	5.98 ± 0.14	5.80 ± 0.00	4.82 ± 0.11
IPLA20018	Babies	F	*Bifidobacterium breve*		0.13 ± 0.00	0.18 ± 0.00	0.70 ± 0.00	5.91 ± 0.01	5.66 ± 0.01	4.54 ± 0.05
IPLA20006	Babies	F	*Bifidobacterium breve*		0.13 ± 0.01	0.24 ± 0.04	0.47 ± 0.01	5.89 ± 0.01	5.53 ± 0.03	4.87 ± 0.01
IPLA20007	Babies	M	*Bifidobacterium longum*		0.06 ± 0.03	0.06 ± 0.02	0.73 ± 0.00	6.18 ± 0.01	5.98 ± 0.01	4.94 ± 0.01
IPLA20011	Babies	F	*Bifidobacterium longum*		0.08 ± 0.01	0.07 ± 0.00	0.56 ± 0.02	6.16 ± 0.01	5.97 ± 0.01	4.95 ± 0.01
IPLA20012	Babies	M	*Bifidobacterium longum*		0.11 ± 0.01	0.11 ± 0.01	0.90 ± 0.01	6.16 ± 0.01	5.97 ± 0.03	4.97 ± 0.01
IPLA20010	Babies	M	*Bifidobacterium pseudocatenulatum*		0.12 ± 0.01	0.15 ± 0.03	0.77 ± 0.00	6.05 ± 0.03	5.85 ± 0.01	4.97 ± 0.00
IPLA20014	Babies	M	*Bifidobacterium pseudocatenulatum*		0.10 ± 0.02	0.13 ± 0.02	0.69 ± 0.03	6.02 ± 0.00	5.84 ± 0.02	4.98 ± 0.00
IPLA20026	Babies	M	*Bifidobacterium pseudocatenulatum*		0.13 ± 0.01	0.18 ± 0.03	0.96 ± 0.02	6.08 ± 0.01	5.79 ± 0.04	5.00 ± 0.01
IPLA20035	Elderly	F	*Bifidobacterium catenulatum*		0.07 ± 0.01	0.09 ± 0.01	0.33 ± 0.01	6.09 ± 0.04	5.77 ± 0.01	5.10 ± 0.01
IPLA20043	Elderly	F	*Bifidobacterium longum*		0.05 ± 0.01	0.08 ± 0.02	0.62 ± 0.05	6.09 ± 0.01	5.86 ± 0.01	4.94 ± 0.00
IPLA20034	Elderly	F	*Bifidobacterium pseudocatenulatum*		0.08 ± 0.02	0.12 ± 0.01	0.99 ± 0.16	6.06 ± 0.01	5.84 ± 0.01	4.97 ± 0.02
IPLA20036	Elderly	F	*Bifidobacterium pseudocatenulatum/catenulatum*		0.10 ± 0.00	0.10 ± 0.00	0.53 ± 0.02	6.07 ± 0.03	5.88 ± 0.00	5.07 ± 0.00
**2´FL degraders**										
IPLA20045	Adults	F	*Bifidobacterium bifidum*	Fast exponential phase	0.00 ± 0.01	0.16 ± 0.02	0.17 ± 0.01	6.44 ± 0.01	5.43 ± 0.01	5.65 ± 0.02
IPLA20049	Adults	NA	*Bifidobacterium bifidum*	Higher exponential and stationary phase	0.01 ± 0.00	0.13 ± 0.00	0.14 ± 0.03	6.45 ± 0.01	5.56 ± 0.02	6.04 ± 0.01
IPLA20048	Babies	M	*Bifidobacterium bifidum*	Higher exponential and stationary phase	0.00 ± 0.00	0.30 ± 0.02	0.43 ± 0.02	6.31 ± 0.00	5.10 ± 0.02	5.14 ± 0.05
IPLA20015	Babies	M	*Bifidobacterium bifidum*	Fast exponential phase	0.02 ± 0.02	0.12 ± 0.00	0.19 ± 0.04	6.32 ± 0.06	5.35 ± 0.04	5.64 ± 0.00
IPLA20024	Babies	F	*Bifidobacterium bifidum*	Higher exponential and stationary phase	0.01 ± 0.00	0.23 ± 0.01	0.28 ± 0.01	6.35 ± 0.01	5.11 ± 0.04	5.21 ± 0.01
IPLA20025	Babies	M	*Bifidobacterium bifidum*	Fast exponential phase	0.00 ± 0.01	0.11 ± 0.02	0.17 ± 0.03	6.31 ± 0.00	5.46 ± 0.02	5.61 ± 0.04
IPLA20017	Babies	F	*Bifidobacterium bifidum*	Fast exponential phase	0.01 ± 0.01	0.38 ± 0.02	0.38 ± 0.04	6.31 ± 0.05	5.72 ± 0.06	5.77 ± 0.01
IPLA20001	Babies	F	*Bifidobacterium longum*	Fast exponential phase	0.07 ± 0.00	0.66 ± 0.00	0.91 ± 0.07	6.06 ± 0.02	4.80 ± 0.00	4.89 ± 0.01
IPLA20002	Babies	M	*Bifidobacterium longum*	Slow 2´FL degrader	0.06 ± 0.01	0.57 ± 0.06	1.11 ± 0.12	5.97 ± 0.01	4.85 ± 0.02	4.80 ± 0.05

IPLA20023	Babies	M	*Bifidobacterium longum*		0.09 ± 0.00	0.67 ± 0.05	0.78 ± 0.08	6.16 ± 0.02	4.91 ± 0.01	4.96 ± 0.01
IPLA20008	Babies	F	*Bifidobacterium longum*		0.07 ± 0.03	0.62 ± 0.04	0.67 ± 0.02	6.19 ± 0.01	4.90 ± 0.01	4.93 ± 0.00
IPLA20037	Elderly	F	*Bifidobacterium bifidum*	Fast exponential phase	0.09 ± 0.12	0.31 ± 0.02	0.27 ± 0.04	6.22 ± 0.22	5.13 ± 0.03	5.15 ± 0.04
IPLA20038	Elderly	F	*Bifidobacterium bifidum*	Higher exponential and stationary phase	0.01 ± 0.02	0.29 ± 0.03	0.26 ± 0.01	6.36 ± 0.01	5.12 ± 0.01	5.15 ± 0.04
IPLA20039	Elderly	F	*Bifidobacterium bifidum*	Higher exponential and stationary phase	0.01 ± 0.01	0.26 ± 0.01	0.29 ± 0.04	6.37 ± 0.01	5.15 ± 0.01	5.18 ± 0.00
IPLA20040	Elderly	F	*Bifidobacterium bifidum*	Higher exponential and stationary phase	0.01 ± 0.01	0.32 ± 0.03	0.26 ± 0.03	6.30 ± 0.04	5.13 ± 0.01	5.14 ± 0.01
IPLA20041	Elderly	F	*Bifidobacterium longum*		0.05 ± 0.01	0.51 ± 0.05	0.54 ± 0.01	6.17 ± 0.03	4.88 ± 0.01	4.94 ± 0.01
IPLA20042	Elderly	F	*Bifidobacterium longum*		0.11 ± 0.00	0.49 ± 0.01	0.57 ± 0.02	6.18 ± 0.02	4.91 ± 0.03	4.94 ± 0.03
R0071	commercial	-	*Bifidobacterium bifidum*	Higher exponential and stationary phase	0.02 ± 0.00	0.71 ± 0.01	0.76 ± 0.09	6.38 ± 0.01	5.04 ± 0.01	4.95 ± 0.01
TMC3115	commercial	-	*Bifidobacterium bifidum*	Fast exponential phase	0.09 ± 0.01	0.30 ± 0.01	0.37 ± 0.01	6.42 ± 0.01	5.17 ± 0.02	5.39 ± 0.02
