# Supplementary figures and images for: 2-Fucosyllactose Metabolism by Bifidobacteria Promotes Lactobacilli Growth in Co-Culture
Source: Microorganisms. 2023 Oct 29;11(11):2659. doi: 10.3390/microorganisms11112659 (PMC10673426; doi:10.3390/microorganisms11112659)

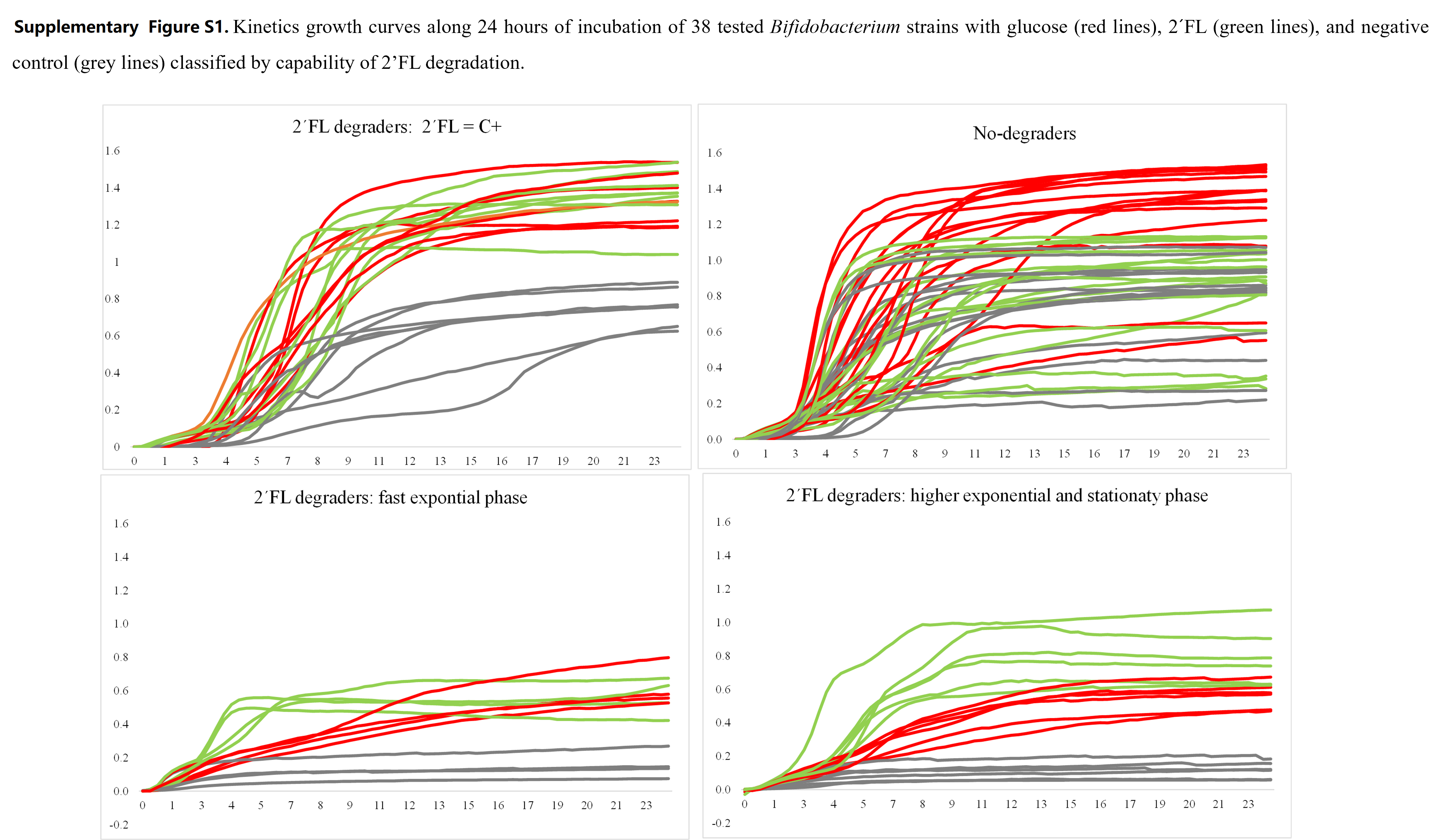

Supplement: Supplementary file 1 [file microorganisms-11-02659-s001.zip › Supplementary Figure S1.tif]
